# Family Members’ Perspectives of Health Care System Interactions With Suicidal Patients and Responses to Suicides: Protocol for a Qualitative Research Study

**DOI:** 10.2196/13797

**Published:** 2019-08-09

**Authors:** Erin Bryksa, Reham Shalaby, Laura Friesen, Kirsten Klingle, Graham Gaine, Liana Urichuk, Shireen Surood, Vincent Agyapong

**Affiliations:** 1 Addiction and Mental Health Alberta Health Services Edmonton, AB Canada; 2 Department of Psychiatry University of Alberta Edmonton, AB Canada; 3 Department of Educational Psychology, Faculty of Education University of Alberta Edmonton, AB Canada

**Keywords:** suicide, family members, public health systems research

## Abstract

**Background:**

Suicide is a major cause of preventable death globally and a leading cause of death by injury in Canada. To support people who experience suicidal thoughts and behaviors and ultimately prevent people from dying by suicide, it is important to understand the individual and familial experiences with the health care system.

**Objective:**

This study aims to explore how suicide victims, and their family members, interacted with the health care system.

**Methods:**

We will invite family members of 6 to 8 suicide victims to participate in the study by sharing their perspectives on both their relative’s as well as their own interactions with the health care system. Interviews will take place in-person and will be audio recorded, transcribed, and analyzed thematically.

**Results:**

The results of the study are expected to be available in 12 months. We expect the results to shed light on the experiences of suicide victims and their family members with the health care system.

**Conclusions:**

Our study results may inform practice, policy, and further research. They may shape how members of the health care system respond to people who are at risk of suicide and their families.

**International Registered Report Identifier (IRRID):**

PRR1-10.2196/13797

## Introduction

### Background

Suicide is a serious global public health problem, with an estimated 800,000 people reported to have died by suicide every year [1]. In Canada, suicide remains the 9th leading cause of death and the 2nd leading cause of death among children, the youth, and young adults [2]. There were a total of 3978 suicides in Canada in 2016 [3], with more than 500 of these deaths occurring in Alberta. This is more than the number of deaths that occurred that year because of motor vehicle collisions [4]. Health care systems play a vital role in suicide prevention. A study in Alberta, for instance, found that the majority of people who died by suicide used a health care service in the year before their death. They were also more likely to use the emergency departments, in-patient services, or community mental health services than those who died from other causes, and typically, they used health care services for mental disorders [5].

In Edmonton, which is the site of this study, suicide prevention initiatives are underway to enhance aspects of the health care system, as evidenced by the Implementation Plan for the Edmonton Suicide Prevention Strategy [6]. Yet, beyond stakeholder engagement [7] and an understanding of the dimensions of service quality [8], little is known locally about how individuals who die by suicide and those close to them experience the health care system. The current mechanism by which the Alberta Health Services (AHS) investigates suicide is through the Quality Assurance Review (QAR). The QAR aims to “assess and evaluate the provision of health care service from a system perspective with the goal of improving the quality of services provided” [9]. Although QARs are done following a suicide, on a case-by-case basis, the results are not shared beyond those directly involved. The privacy of the QAR limits case comparison and knowledge translation. In addition, an understanding of the context of death by suicide is needed, as it is thought to differ from the context of a suicide attempt. In 1 study, although the groups were generally the same on measure of depression, completers were significantly more likely to have experienced significant job stress and financial problems, left a suicide note, and used alcohol and drugs before the act [10]. The AHS is committed to patient- and family-centered care [11], which highlights the importance of talking to families about their own and their relatives’ experiences with the health care system. Ultimately, insight into the experiences of people who die by suicide and their family members has the potential to inform policy and practice in shaping how members of the health care system, and the AHS specifically, respond to individuals who are at risk of suicide.

### Objective and Research Questions

The purpose of this study was to better understand how suicide victims and their family members interacted with the health care system. Our specific research questions include the following: (1) How do individuals impacted by the suicide of a family member perceive their relative’s interactions with the health care system? (2) How do individuals impacted by the suicide of a family member perceive their own interactions with the health care system?

### Conceptual Framework

The conceptual framework is based on empirical evidence, suggesting that it is not only individual and family and societal factors that contribute to deaths by suicide but also factors related to health care systems (as shown in Textbox 1 [1,12-35]). The framework has been developed through a general review in the literature aimed at providing an evidence-based material supporting all possible risk factors for suicide. Although focusing on health care system risk factors alone would clearly support our hypothesis, we opted to conceptualize the study based on all possible risk factors, including family and social factors such as stigma, social status or public education [1,12], as well as individual factors such as gender, prior history of attempted suicide or psychiatric illness [1,13-15], which we expect to be identified by our participants throughout the data collection. This work would subsequently help in providing a framework for formulating a supplementary quantitative study, which would use descriptive and inferential statistical analysis to explore risk factors for suicide in a provincial or national sample of family members who have been impacted by the death of a loved one through suicide.

Findings from the study will illuminate strengths of the health care system and aspects of care in need of further improvement and refinement. The recommendations arising from this study have the potential to lead to significant system enhancements and reduce suicide rates in Alberta and beyond.

**Conceptual framework of factors contributing to deaths by suicide. The conceptual framework was developed by the authors based on existing literature.**

Health systems factors

- Staff attitude toward suicidal persons [12]
- Recency of hospitalization for attempt suicide and recent health care contact [12,13,16,21,30]
- Underdiagnoses of mental disorders and major depressions [31]
- Brevity of interaction with the medical staff [32]
- Ignoring the warning signs of suicide by the health care providers [33]
- Lack of trust in the health care services [34]
- Relatives feeling of exclusion from treatment information [33]

Family/societal factors

- Higher versus low- and middle-income countries [1]
- Stigma and taboo [1]
- Public education [12]
- Interpersonal problems [19]
- Family positive/negative life events and social support [22,23]
- Family history of suicide [24,27]
- Familial difficulty in seeking help outside the social network [35]

Individual factors

- Prior history of attempted suicide [1]
- Psychiatric illness [13-15]
- Gender [1,17]
- Race/Ethnicity [17]
- Age [17]
- Relationship problems/losses [18]
- Recent/impending crisis [18]
- Education level [19]
- Alcohol/substance abuse [19,28,29]
- Marital status [20]
- Hopelessness [25]
- Vulnerable groups with discrimination experience [1,26]

## Methods

### Study Design

This study uses qualitative research design with key informant interviews where sample sizes are kept small to allow for in-depth exploration of phenomena [36]; this also takes into consideration the laborious nature of case-by-case analysis [37]. We will use the interpretative phenomenological analysis (IPA). IPA makes sense of the participant’s experiences by seeking to understand the cognitive, affective, linguistic, and physical being while maintaining the researcher as an integral part of the sense-making process [37,38]. This methodology is best used in studies where the objective is to explore the meaning behind the experiences of participants. IPA is an especially fitting research approach when the researcher intends on asking complex, broad, and open-ended questions [37], as is the case in this research proposal. In keeping with the open-ended nature of qualitative research, this method allows the researcher to explore the research question in a flexible nonprescriptive manner, thus facilitating a more thorough exploration [38]. Although quantitative methodologies can supply information on the *what* aspect of the phenomenon, they do not aim to develop understandings about personal experiences and *why* and *how* phenomena occur. The choice to use qualitative methodology for this research was based on the need to better understand the interactions of important factors related to the phenomenon under study [39]. The methodology was also chosen because there is a need to better understand how the phenomenon is being experienced and understood by those who go through it personally. Quantitative methodologies, although valuable, are not appropriate to explore how individuals experience a phenomenon. Statistical methods may provide an estimate about how many individuals have been impacted by suicide or how many individuals who have suicidal ideation access the health care system, but they do not explain how suicide impacts different individuals personally and how different factors interact. The explanations happen through the interpretation of the data. As stated, statistical data do not shed light on the *why* or *how* of situations. A common example used to caution against interpreting correlations is as follows: “there is a correlation between eating ice-cream and drownings.” One individual might say, “eating ice-cream causes drownings!” Another would say, “in the summer-time, more individuals like to eat ice-cream. People also tend to swim more in the summer and may drown. Perhaps someone ate a lot of ice-cream before swimming and as we know, eating immediately before swimming may increase your risk of drowning.” Although researchers can theorize about interpretations of quantitative results, proponents of qualitative research hold that it is best to go to the sources and ask for explanatory stories in an explorative manner.

### Ethics and Operational Approval

The study will be conducted in accordance with the Declaration of Helsinki (Hong Kong Amendment) and Good Clinical Practice (Canadian Guidelines). Written informed consent will be obtained from each participant. The study has received ethical clearance from Health Ethics Research Board of the University of Alberta (reference number Pro00086420)**.**

### Participants

About 6 to 8 key informants will be recruited to participate in the study through purposive sampling.

Participants who meet the following criteria will be approached and invited to participate in the study: aged 18 years or older; English speaking; have the ability to give informed consent; have a close family member who has died by suicide (eg, spouse, parent, child, sibling, and grandparents); one member per family of suicide victim for interviews; a minimum of 3 months has passed since their family member has died to avoid the acute bereavement period; had regular contact with their family member, before their suicide, such that they are reasonably aware of their interaction with the health care system.

### Recruitment

The following steps outline how participants will be identified for the study:

- Recruitment poster (Multimedia Appendix 1) would be posted in all the recruitment sites, which directs participants to the research coordinator, should they express interest in participating in the study.
- Posters would be shared mainly with the Canadian Mental Health Association (CMHA) in Edmonton Zone.
- The Executive Director for the Edmonton Zone (CMHA) will distribute the recruitment poster to their contacts, including groups run by the organization, and potentially use social media as a platform to recruit, in Alberta.
- The recruitment poster will also be shared with the Centre for Suicide Prevention for distribution to their contacts and on social media.
- The researcher coordinator will promote the study at a Suicide Prevention and Awareness Booth at a Mental Health Walk in Edmonton in June 2019.
- This process will continue until 6 to 8 participants have been recruited.
- During analysis of the data, if saturation has not been achieved, we will open up the recruitment until fulfilling saturation.

### Data Collection

The key informant interviews will comprise an in-person semistructured interview with each participant. Interviewers will be graduate students trained in qualitative interviewing and will be supervised by the research coordinator. The interview will span 1.5 to 2 hours and will allow for a certain degree of flexibility in the types of questions asked, thus providing rich and contextualized understanding while also creating some consistency across participant interviews. More specifically, an interview protocol has been developed, and interviewers will aim to ask all the questions. However, they may change the order of the questions if participants lead the conversation in different ways. Interviewers may also prompt individuals when needed with statements such as “tell me more” and “can you tell me a story about a time when…” To facilitate this exploration, we will adopt what is known as a *funneling technique* to sequence the topics covered, starting first with the more general topics and then moving to the more salient or emotionally laden topics [37]. Using this technique, the interview will unfold in a logical and relaxed fashion while taking into consideration the sensitive nature of the subject matter. Saturation will be reached when no new information is added to the interview. Data saturation can be conceptualized in different ways: individual-level data saturation and group-level data saturation. During the individual interviews, the researcher needs to continue to probe until they feel they have a good understanding of the individual’s experiences; this is one level of saturation. During the group-level analysis, saturation is considered to be reached when no new research codes (or themes) are being developed when nothing new is coming up to support a concept. When the information that researchers are getting from participants is becoming repetitive or redundant, then researchers argue that data saturation has been reached [40]. Both levels of saturation will be considered in the research process.

The interview will be audio recorded and then later transcribed verbatim. Interviews will take place in 3 main AHS sites according to the patient preference. Data from all participants will be compared and contrasted only after each participant’s experience has been analyzed and considered on its own.

### Data Analysis

The primary aim of data analysis will be to discover common themes according to individual experience and across participants. In keeping with traditional forms of content analysis, we will begin by reading and rereading the transcribed interviews, known as the preliminary exploratory analysis phase [41], while considering possible concepts or themes. The purpose of this phase is to begin to develop a sense of the data as a whole. We will then begin coding the data, a process whereby the researcher makes sense of the data by dividing text into sections. We will initially code the transcript data at a relatively low level of abstraction by looking for common concepts as seen in individual interview data as well as across participants. We will proceed to a higher level of abstraction by grouping the codes into common themes and subthemes, a process known as *clustering* [37]. This process is iterative and involves many revisions, checking back to ensure that the proposed themes fit with the transcription data. Themes will then be inputted in a table, tracking the overarching theme(s), subordinate themes, and where the theme emerges in the transcribed data (ie, page number). From this point, certain themes will be selected based on importance (how the theme adds to the overall account) and how it adds to the richness of data. The final stage of data analysis will include writing up the themes in the form of a narrative using transcribed data to account for and justify the coding of certain themes. The final analysis will be broken into 2 sections: the findings, where a description of each theme and the corresponding quotes will be presented, and the discussion section, where we will interpret the findings in light of the extant literature. The demographic data to be collected as part of the interview would identify the relationship between the study participants and the persons who died by suicide. These data would allow for the identification of responses and matching as well as comparison with potential confounding factors such as age, sex, and relationship

### Evaluation of the Goodness of Qualitative Research

Credibility and confidence are essential considerations when addressing the area of qualitative enquiry. This is because qualitative enquiry is intrinsically interpretive, and the investigators are as much a part of the study as the participants [41]. For the purpose of this study, and similarly to our approach in a concurrent borderline personality disorder study being undertaken by the team, we will enhance credibility and confidence by way of member checking and external appraisals. Member checking necessitates the investigator check with participants with regard to the validity of findings by asking clarifying questions used to enhance and deepen the interpretations, as they arise in the interview with the participant. External appraising considers the estimations of another person, for instance, a coinvestigator or research supervisor, aimed at reviewing codes and themes in search of continuity and validity [41]. The study findings will also be thoroughly and carefully evaluated for consistency, persuasiveness, comprehension, and practicality [42,43].

## Results

We anticipate that recruitment for the study would begin in April 2019, and we expect the study findings to be available within 12 months. We expect the findings to shed light on the experiences of suicide victims and the experiences of their family members with the health care system. The findings will be disseminated at several levels, including patients, family members, practitioners, academics, researchers, and health care organizations.

## Discussion

### Expected Findings and Implications

Both those who experience suicidal ideation and those close to them can offer their perspectives into the experience. When a relative dies by suicide, family members can offer valuable insight into their relatives’ interaction with the health care system. Similarly, understanding family members’ own experiences with the health care system is also valuable. In a previous study, family members noted that the most significant barrier to identifying and managing suicide in primary care is the brevity in interactions with physicians [32]. In another study, family members reported that despite the presence of warning signs, their relatives were overlooked by health care providers in the lead-up to their suicide [33]. Another study of elderly people who died by suicide found them to have a general distrust in the health care service [34]. Despite the negative views expressed in these studies about the health care system, one study reported on the compassionate care their relative received by the health care providers [32], thus underscoring the potential for health care interactions to serve as a means of support among suicidal individuals. Reflecting on their relatives’ death by suicide would understandably highlight the perceived inadequacies and potential strengths of the health care system. Family members may feel excluded from treatment information [33] and experience difficulty in seeking help outside the social network [35]. This is especially poignant, given that family members may continue to interact with the health care system after the death of their loved one [44-47]. Although it is positive to see that family members are consulted, there remain gaps in how patients and families perceive their care and the impact it may have on their health outcomes. What appears to be needed is an understanding of the experiences of death by suicide in the context of the local health care systems. In light of the above, family members who share their perspectives on their relatives’ interaction with the health care system, and their own, offer valuable insight, which could potentially inform system policy and enhancements as well as reduce suicide rates in Alberta and beyond.

### Conclusions

Our study would be the first in Alberta to examine how suicide victims and their family members interacted with the health care system. Our study results may inform practice, policy, and further research in Alberta, Canada, and internationally. They may shape how members of the health care system respond to people who are at risk of suicide and their families.

## Appendix

Multimedia Appendix 1 Recruitment poster. Participants needed: adults who have lost a family member to suicide.

## Abbreviations

AHS: Alberta Health Services
CMHA: Canadian Mental Health Association
IPA: interpretative phenomenological analysis
QAR: Quality Assurance Review
