# Supplementary material for: Family Members’ Perspectives of Health Care System Interactions With Suicidal Patients and Responses to Suicides: Protocol for a Qualitative Research Study
Source: JMIR Res Protoc. 2019 Aug 9;8(8):e13797. doi: 10.2196/13797 (PMC6709894; doi:10.2196/13797)
Supplement: Multimedia Appendix 1 [file resprot_v8i8e13797_app1.pdf]

PARTICIPANTS NEEDED: Adults who have lost a family member to suicide

Family Members' Perspectives of System Responses to Suicides

We are looking for volunteers living within Edmonton Zone, to share their stories about how their family member who died by suicide, and themselves, interacted with the health care system.

Are you someone who:

- Has a family member who has died by suicide more than 3 months ago?
- Knows that your family member interacted with the health care system (e.g., family doctor, emergency department, hospital inpatient, outpatient services, etc.)
- Had regular contact with your family member so that you are aware of their interaction with the health care system?
- Is 18 years or older?

Being in this study is your choice. If you choose to participate, you will be asked to take part in one in-person interview approximately 1.5 to 2 hours long. The interview will be in Edmonton.

By taking part in this study, you will help us to identify strengths, areas of improvement and ideas for change for our health care system.

To learn more about this study, or to participate, please contact the research coordinator:

Laura Friesen (Research Coordinator)	Email: laura.friesen2@ahs.ca Telephone: 780-342-7965

Be heard. Your stories matter and can make a difference!

The plan for this study has been reviewed for its adherence to ethical guidelines by a Research Ethics Board at the University of Alberta.
